# Tumor necrosis factor-α (TNF-α)-308G/A promoter polymorphism in colorectal cancer in ethnic Kashmiri population — A case control study in a detailed perspective

**DOI:** 10.1016/j.mgene.2016.06.001

**Published:** 2016-06-03

**Authors:** Mujeeb Zafar Banday, Henah Mehraj Balkhi, Zeenat Hamid, Aga Syed Sameer, Nissar A. Chowdri, Ehtishamul Haq

**Affiliations:** a Department of Biotechnology, University of Kashmir, Hazratbal, Srinagar, Kashmir, India; b Department of Basic Medical Sciences, College of Medicine, King Saud Bin Abdulaziz University for Health Sciences, Jeddah, Saudi Arabia; c Department of Surgery, Sher-I-Kashmir Institute of Medical Sciences, Soura, Srinagar, Kashmir, India

**Keywords:** Colorectal cancer (CRC), Kashmir, Case control study, Tumor necrosis factor-α (TNF-α), Polymorphism, Single nucleotide polymorphism (SNP)

## Abstract

**Background:**

Inflammation constitutes one of the important components of colorectal cancer (CRC) pathogenesis. Tumor necrosis factor-α (TNF-α), a cytokine and an important inflammatory mediator plays a pivotal role in the malignant cellular proliferation, angiogenesis, tissue invasion and metastasis in CRC. The studies on association of various polymorphisms in human TNF-α gene including TNF-α-308G/A single nucleotide polymorphism (SNP) are limited, mixed and inconclusive.

**Materials and methods:**

The aim of this study was to analyze the association of TNF-α-308G/A promoter SNP with colorectal cancer (CRC) susceptibility and development risk and also to evaluate the modifying effects of possible TNF-α-308G/A genotypes on different risk factors of CRC in ethnic population of Kashmir, India through a case–control setup. The genotype frequencies of TNF-α-308G/A promoter SNP were compared between 142 CRC patients and 184 individually matched healthy controls by using polymerase chain reaction and restriction fragment length polymorphism (PCR-RFLP) method. The associations between the TNF-α-308G/A SNP and CRC risk were examined through conditional logistic regression models adjusted for multiple possible confounding (third) variables. Further, the associations between this SNP and various clinico-pathological parameters, demographic variables and environmental factors within the case group subjects with regard to CRC risk were also evaluated.

**Results:**

The association between the TNF-α-308G/A SNP and the modulation of risk of CRC was not found to be significant (p value = 0.156). The effect of less common TNF-α-308A allele on the risk of colorectal cancer was also not found to be significant (p value = 0.175). The variant genotype (AA) was nonexistent in the study population. Further, we found no significant effect modulation of CRC risk by wild and heterozygous TNF-α-308G/A SNP genotypes in presence of different possible risk factors (p > 0.05). We also found no significant association of TNF-α-308G/A SNP with the subsets of various characteristics of the case group subjects under study (p > 0.05).

**Conclusions:**

This study indicates that there is no significant association between the TNF-α-308G/A promoter SNP and the risk of developing CRC in ethnic Kashmiri population. However, in order to substantiate our findings, this study needs to be replicated with bigger sample size and should involve other ethnically defined populations with high CRC risk.

## 1 Introduction

Colorectal cancer (CRC) defined as the cancer of the colon, rectum and appendix is the third most common cancer in the world and the fourth leading cause of cancer-related mortality worldwide (Jemal et al., 2011). Globally, it ranks third amongst the cancers in men and second in women (Torre et al., 2016). The incidence of this cancer varies greatly across different geographic locations and among different racial or ethnic groups worldwide and also within a country with populations of multiple races or ethnic groups. In Kashmir valley, CRC is the third most common gastrointestinal (GIT) cancer (Sameer, 2013a, Sameer, 2013b) and is the third most common cancer amongst the women and fourth amongst the men (Wani et al., 2014, Sameer, 2013a).

The CRC development is influenced by or contributed to by multiple factors that include dietary and lifestyle habits, demographic factors, various pathological conditions and genetic predispositions (Arem et al., 2013, Otani et al., 2003). Each of these factors may act alone or in combination with others in modulating the risk of development of CRC (Nieminen et al., 2014, Yehuda-Shnaidman and Schwartz, 2012). Several studies show a clear association between inflammatory processes and also of genetic predispositions particularly those associated with inflammation and the risk of development of CRC (Garrity-Park et al., 2012, Li et al., 2011, Nieminen et al., 2014, Wang and DuBois, 2013, Yehuda-Shnaidman and Schwartz, 2012). These studies along with several others clearly indicate that the inflammation as a whole including the processes and modulators associated with it together with genetic factors particularly those associated with inflammation and its modulators play a critical role in the development of CRC. Several studies now indicate that chronic inflammation increases the risk of development of various cancers including CRC and contributes directly to tumor development mainly through the recruitment of inflammatory cells for precancerous functions (Coussens and Werb, 2002, Mantovani et al., 2008, Pasche, 2013, Zumsteg and Christofori, 2009).

Cytokines play a key role in the mediation and regulation of immune response including inflammation. Several of these cytokines including pro-inflammatory and anti-inflammatory have been implicated in the process of tumorigenesis (Dranoff, 2004). Tumor necrosis factor-α (TNF-α) is a predominantly pro-inflammatory, pleiotropic cytokine produced mainly by activated macrophages and also by several other cell types that include lymphocytes (T cells and natural killer (NK) cells), neutrophils, eosinophils, mast cells, endothelial cells, fibroblasts, and neurons (Schwabe and Brenner, 2006). TNF-α plays a key role in several vital processes including inflammation, autoimmunity, cell growth, proliferation, differentiation and apoptosis (Aggarwal et al., 2012, Beutler and Bazzoni, 1998, Esposito and Cuzzocrea, 2009, Waters et al., 2013). It is involved in defense against viral infections, mediates the early inflammatory and acute-phase responses and play vital immune-regulatory activities through the production of other cytokines and adhesion molecules such as endothelial adhesion molecules (Seo and Webster, 2002, Strieter et al., 1993).

Several studies have reported the involvement of TNF-α and the important role it plays in the pathogenesis and progression of various cancers (Aggarwal et al., 2012, Moore et al., 1999, Shishodia et al., 2003, Wang et al., 2009, Wilson et al., 1995). TNF-α has been shown to stimulate malignant cellular proliferation; angiogenesis, through the induction of endothelial cell proliferation and by modulating the expression of various pro-angiogenic factors (Leek et al., 1998); tissue invasion and metastasis by inducing the expression of pro-migratory adhesion molecules (Balkwill, 2009, Ioculano et al., 1995, Kirkpatrick et al., 2004, Shishodia et al., 2003) and also induce the inflammatory pathways that enhance tumorigenesis (Szlosarek and Balkwill, 2003). The expression of TNF-α like other cytokines is tightly regulated at the transcriptional level and also at post-transcriptional level. The polymorphisms located within the regulatory regions of various cytokine genes including TNF-α have been reported to influence the expression and secretion of these cytokines (Raabe et al., 1998, Wilson et al., 1997). Several single-nucleotide polymorphisms (SNPs) in human TNF-α gene have been reported and most of these polymorphisms are found within the promoter region of the gene and are located at positions − 1031, − 863, − 857, − 851, − 575, − 376, − 308, − 244, − 238, + 70, and + 71 nucleotides (nt) relative to the start site of transcription (Baena et al., 2002, Bidwell et al., 1999, Marsh et al., 2003, Wang et al., 2011).

The functional significance of many of these SNPs such as those at positions − 238, − 308, − 376, − 857, − 863 and − 1031 has been reported and elucidated and have been related to the modulation of TNF-α production mostly through their effect on transcriptional activity that in turn results in differential gene expression (Hellmig et al., 2005, Wilson et al., 1997). Among these polymorphisms, the biallelic SNP in the promoter region at position 308 bp upstream of the TNF-α transcriptional start site representing guanine (G) to adenine (A) substitution (TNF-α-308G/A; rs1800629) has been extensively studied and is one of the most common TNF-α SNPs in general populations (Hajeer and Hutchinson, 2001). The more common − 308G allele is sometimes referred to as TNF1 or 308.1 whereas the less common − 308A allele is referred to as TNF2 or 308.2. TNF-α-308A allele has been associated with enhanced baseline/constitutive and inducible TNF-α expression (Kroeger et al., 1997, Zhuang et al., 2013) both in vivo and in vitro (Kroeger et al., 2000, Louis et al., 1998) and is associated with increased plasma levels of TNF-α in comparison with TNF-α-308G allele (Wilson et al., 1992) and same is supported by gene reporter assays (Wilson et al., 1997).

Various meta-analyses and other studies have reported the association of TNF-α-308G/A polymorphism with differences in susceptibility between different individuals and on a larger scale between different populations to several diseases including various types of neoplastic diseases (Campelo et al., 2007, Ho et al., 2004, Plevy et al., 1996). Several studies performed in different populations worldwide have demonstrated the association of TNF-α-308A allele related higher expression levels of TNF-α with susceptibility to, progression, metastasis, prognosis, survival and overall outcome of several types of malignant human neoplasms and have established it as a putative risk factor for developing immune related and otherwise, malignant neoplastic disease including colorectal cancer. The various malignant disorders studied with regard to TNF-α-308G/A SNP include oral, head & neck cancers (Correa et al., 2011, Gupta et al., 2008); gastric, lung and bone cancers (Gorouhi et al., 2008, Hong et al., 2013, Patio-Garcia et al., 2000, Wang et al., 2011, Xie et al., 2014); prostrate, breast, cervical and uterine endometrial cancers (Jin et al., 2014, Ma et al., 2014, Sasaki et al., 2000, Wang et al., 2011, Zhang and Zhang, 2013); hepatocellular, bladder and renal cell carcinomas (Marsh et al., 2003, Nakajima et al., 2001, Nonomura et al., 2006, Yang et al., 2011a); non-Hodgkin lymphomas (Zhai et al., 2014); esophageal cancer (Guo et al., 2005) and in particular here CRC (Chen et al., 2013, Garrity-Park et al., 2008, Garrity-Park et al., 2012, Li et al., 2011, Min et al., 2014).

In this study, we systematically conducted a case–control evaluation of the possible association between TNF-α-308G/A SNP and susceptibility to CRC in Kashmiri population. We also evaluated the possible effect modulation of CRC risk by age, gender and smoking status. Further, we investigated the possible relationship of this SNP with various clinico-pathological parameters, demographic variables and environmental factors including smoking habit and hence their role in modulating the risk of colorectal cancer in the population under study.

## 2 Materials and methods

### 2.1 Study subjects

The present study involved two subject groups: case and control. The case group included 142 consecutively recruited patients with histopathologically confirmed primary colorectal cancer who underwent surgical resection for primary CRC tumors at the Department of General Surgery, Sher-I-Kashmir Institute of Medical Sciences, the largest and the only tertiary care hospital in the Kashmir valley. The tumor stage and tumor grade were classified according to the 8th edition of TNM classification of Union International Control of Cancer (UICC). Only those cases who had not received any chemo or radiotherapy were chosen for this study. All the cases were more than 18 years old and had no prior history of any malignancy. Blood and tissue samples were obtained from these CRC patients. The control group included 184 healthy individuals with no history or prior diagnosis of any malignant disorder or any other serious disease from whom blood was collected and used as control for the present study. The control group included both population based subjects and hospital-based subjects. The control group subjects were matched to the case group subjects individually for age (± 5 years), sex, place of residence (rural/urban) and smoking habit. Both the case and control subjects chosen for this study were ethnic Kashmiris.

### 2.2 Data collection

The data relevant to the study concerning all the CRC patients including various clinico-pathological parameters, demographic variables and environmental factors including smoking habit were obtained and evaluated from patient medical records (files), pathology reports and also from the personal interviews with patients and/or their guardians (for those who were illiterate). The data collected included tumor location, Dukes Stage, lymph node status, sex, age, place of residence, smoking habits among several other relevant parameters. The relevant data was also obtained for each of the recruited controls mostly through personal interviews. All the patients and/or their guardians were informed about the study and their willingness to participate in this study was documented using a predesigned questionnaire. Same procedure was followed for the controls. The study was approved by the Institutional Ethics Committee. The work was carried out in accordance with The Code of Ethics of the World Medical Association (Declaration of Helsinki) for experiments in humans.

### 2.3 Sample preparation and DNA extraction

The tumor tissue samples collected after surgical resection were immediately snap frozen in liquid nitrogen and then stored at − 80 °C until further use for DNA extraction and other research purposes. Prior to DNA extraction, tumor tissue samples were washed 2–3 times in phosphate buffered saline (PBS) and adipose and connective tissue portions if any were dissected away. Peripheral blood sample, 3–5 mL from each case and control group individual was collected into EDTA-blood vacutainer collection tubes and stored at − 80 °C until further use. Genomic DNA was extracted from both the tumor tissue and blood specimens using DNeasy Blood and Tissue Kit (Qiagen, Germany) and Quick-gDNA™ MiniPrep kit (Zymo Research, US) according to the manufacturers' instructions. The extracted DNA was stored at − 20 °C until further use. The qualitative and the quantitative assessments of the extracted genomic DNA samples were carried out by absorbance measurements at 260 nm and 280 nm using UV–visible spectrophotometeric analysis and also by agarose gel electrophoresis. The DNA extracted from blood samples of case and control group subjects was used for this study.

### 2.4 Single nucleotide polymorphism (SNP) analysis or genotyping

The TNF-α-308G/A (rs1800629) SNP was genotyped using polymerase chain reaction-restriction fragment length polymorphism (PCR-RFLP) assay.

### 2.5 TNF-α-308G/A PCR

The PCR for TNF-α-308G/A SNP was carried out in a total volume of 25 μL containing 100 ng–1 μg of genomic DNA, 0.7-1 U Taq DNA polymerase with lX Standard Taq reaction buffer (New England Biolabs, UK), 2.1 mM MgCl_2_; 0.28 mM deoxynucleotide triphosphate mix (New England Biolabs, UK); 0.56 μM forward and revere oligonucleotide primers (Integrated DNA Technologies, India) and nuclease-protease free water (Qiagen, Germany) added up to a final volume of 25 μL.

**Fig. 1:**
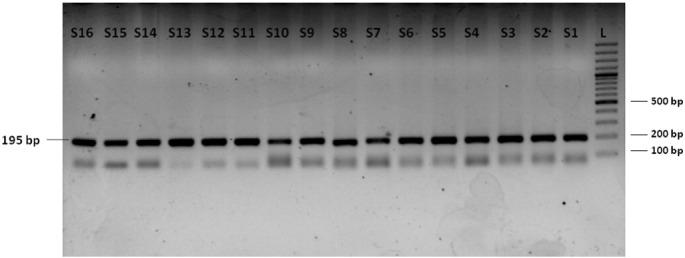
Electrophoresis of TNF-α-308G/A SNP PCR products on a 2.5% agarose gel. Lanes S1–S16: amplified PCR products with prominent/desired band 195 bp in size. Lane L: 100 bp molecular size marker/ladder.

The PCR conditions used for the amplification of TNF-α region encompassing − 308G/A SNP were as follows; initial denaturation at 95 °C for 6 min followed by 35 cycles of denaturation at 95 °C for 45 s; annealing at 65 °C for 60 s and extension at 72 °C for 60 s followed by a single final extension step at 72 °C for 10 min. The oligonucleotide primers used for this amplification were 5′-GGAGGCAATAGGTTTTGAGGGCCAT-3′ (forward) and 5′-CTGTCT-CGGTTTCTTCTCCATGGCG-3′ (reverse). The underlined base at the 3′-end of the forward primer was incorporated to generate an *Nco*I restriction site in the polymorphic region under study (Wilson et al., 1992). The desired PCR product obtained for TNF-α-308G/A SNP was 195 bp in size (Fig. 1).

### 2.6 Genotyping

**Fig. 2:**
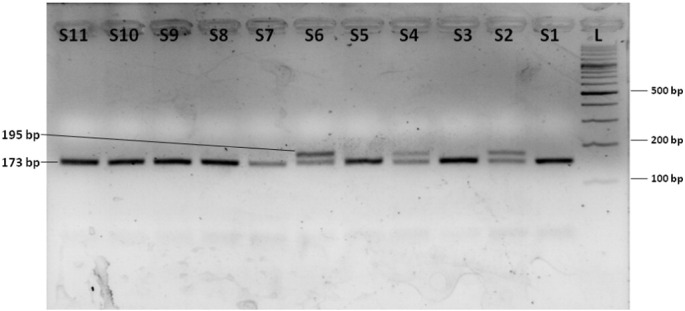
Electrophoresis of TNF-α-308G/A SNP genotyping by PCR-restriction fragment length polymorphism on a 4% agarose gel. Lanes S1–S11: restriction digestion products; wild genotype (GG) is cleaved by *Nco*I enzyme yielding two fragments of size 173 bp and 22 bp while as variant genotype (AA) yields a single undigested fragment of 195 bp. Heterozygous genotype (GA) yields three fragments 195 bp, 173 bp and 22 bp in size. The 22 bp fragment is not visible in the picture. Lanes S2, S4 and S6 show the heterozygous genotype (GA) while as rest of the lanes show wild genotype (GG) of TNF-α-308G/A SNP. Variant genotype (AA) of TNF-α-308G/A SNP was not observed in any of the samples studied. Lane L: 100 bp molecular size marker/ladder.

The TNF-α-308G/A SNP was genotyped using the restriction enzyme *Nco*I (Thermo Fisher Scientific, US). The digestion was carried out according to the manufacturers' instructions in a 30 μL reaction volume containing 10 μL of PCR product and 10 U of *Nco*I enzyme and incubated at 37 °C overnight. *Nco*I enzyme cleaved the TNF-α-308G (TNF1) allele into two fragments of 173 bp and 22 bp but not the TNF-α-308A (TNF2) allele that gave a single undigested fragment of 195 bp. The digestion products were separated on 3.6–4.0% agarose gel stained with ethidium bromide (Fig. 2).

The genotyping errors including the false estimates of a particular allele frequency and to check the reproducibility of the genotyping done, 10% of case and control samples selected randomly were re-genotyped. In addition, in each PCR-RFLP setup, previously amplified and genotyped samples representing all possible case scenarios were included as a reference control (Bonin et al., 2004, Wang et al., 1998).

## 3 Statistical analysis

The effective sample size and statistical power were computed by using “Genetic Power Calculator” developed by Purcell et al. (http://pngu.mgh.harvard.edu/~purcell/gpc/). The statistical power of 80% is used widely to avoid false negative associations and to determine a cost-effective sample size under the assumption of 25% variant allele frequency, 1:1 case-to-control ratio, and 5% type I error rate (α).

The numbers and percentages were calculated and presented for each of the categorical variables along with means, standard deviations (SD), median and inter-quartile range for continuous variables. Conditional logistic regression analysis was carried out to calculate unadjusted and adjusted odds ratios (ORs) and corresponding 95% confidence intervals (CIs) to assess the possible association of relevant TNF-α-308G/A SNP genotypes with CRC risk and to assess the possible gene-environment interactions. In order to eliminate the possible confounding (third) variables, the conditional logistic regression models were adjusted for known risk factors like gender, age and smoking habit and also with place of residence. The comparison of genotype and allele distributions of TNF-α-308G/A SNP between the CRC patient group and control group using conditional logistic regression involved two-sided chi-square test. The correlation between genotypes and clinico-pathological parameters, demographic variables and environmental factors including smoking habit within the case group was analyzed by Fisher exact test. The fitness of the genotype distributions to Hardy-Weinberg equilibrium for allele and genotype frequencies in the population under study was tested using the chi–square test. A two sided probability value of or less than 5% (p ≤ 0.05) was considered statistically significant for all types of analyses. All statistical analyses were performed using IBM SPSS Statistics v21 software.

## 4 Results

A total of 142 primary CRC patients and 184 control subjects were included in this study with prior consent of each individual. The frequencies of various clinico-pathological parameters, demographic variables and environmental factors in colorectal cancer case subjects and relevant parameters in control subjects are listed in Table 1. The case group consisted of 59.86% (85/142) males and 40.14% (57/142) females (male/female ratio = 1.49); 53.52% (76/142) subjects were > 50 years old and 46.48% (66/142) subjects were ≤ 50 years of age. The mean age of the case group subjects was 52.68 years; for males it was 54.70 years and for females it was 50.66 years and the age range was 21–82 years. The control group consisted of 55.43% (102/184) males and 44.57% (82/184) females (male/female ratio = 1.24); 50.54% (93/184) subjects were > 50 years old and 49.46% (91/184) subjects were ≤ 50 years of age. The mean age of the control group subjects was 52.22 years; for males it was 53.64 years and for females it was 50.80 years and the age range was 21–80 years. The difference in the distribution of gender and age among the case group and control group subjects was not statistically significant (p > 0.05) (Table 1). Among the case group subjects, 61.27% (87/142) were rural residents and 38.73% (55/142) were urban residents and among the control group, 54.89% (101/184) subjects resided in rural areas and 45.11% (83/184) subjects resided in urban areas. Further, the case group consisted of 56.34% (80/142) smokers and 43.66% (62/142) non-smokers while as the control group consisted of 51.09% (94/184) smokers and 48.91% (90/184) non-smokers. No statistically significant dwelling and smoking status related differences were observed between the case and control group subjects (p > 0.05) (Table 1).

The frequencies of the genotypes for TNF-α-308G/A SNP for both the case and the control groups are listed in Table 2. The more common GG genotype of TNF-α-308G/A SNP was more frequent among the case group [87.32% (124/142)] in comparison to the control group [81.52% (150/184)].The frequency of the heterozygous genotype (GA) was less in the case group [12.68% (18/142)] than in the control group [18.48% (34/184)]. The TNF-α-308G/A SNP variant genotype (AA) was not found in either of the two study groups (case and control). The overall association between the TNF-α-308G/A SNP and the modulation of the colorectal cancer risk in the population under study was found to be non-significant (p > 0.05). Notably, we didn't find the TNF-α-308G/A SNP variant genotype (AA) in our population. Further, the frequency of the more common TNF-α-308G allele was found to be 93.66% (266/284) among the case group subjects and 90.76% (334/368) among the control group individuals. The frequency of the less common TNF-α-308A allele was found to be 6.34% (18/284) among the case group subjects and 9.24% (34/368) among the control group individuals. This distribution of alleles among the case and control groups was not found to be significantly different (p > 0.05). Further, the genotype frequencies for TNF-α-308G/A SNP among both the case and control groups were found to be in agreement with Hardy-Weinberg equilibrium (HWE) (cases: χ^2^ = 0.65; p value = 0.420 and controls: χ^2^ = 1.91; p value = 0.167).

The possible effect modulation of CRC risk by age, gender and smoking status is summarized in Table 3. We did not observe any significant effect modulation by wild and variant TNF-α-308G/A SNP genotypes in presence of these different possible risk factors (p > 0.05).

The number and the frequencies of the subsets of various characteristics of the case group subjects under study i.e. age, gender, dwelling, smoking status, tumor location, tumor grade and lymph node status for the TNF-α-308G/A SNP are listed in Table 4. We also analyzed the correlation of this TNF-α SNP with the subsets of these various characteristics of the case group subjects but did not find any characteristic subset to be significantly associated with the genotypic status of the TNF-α-308G/A SNP (p > 0.05).

## 5 Discussion

CRC is one of the most prevalent cancers worldwide and is associated with a high degree of cancer related mortality and morbidity. CRC is one of the few cancers in which inflammation is mostly evident at the earliest stages of tumor progression as a basic pathological incident and is capable of transforming the incipient tumors into full blown malignancy (Mantovani et al., 2008, Qian and Pollard, 2010, Slattery and Fitzpatrick, 2009, Wogan et al., 2012). CRC development has a strong association with innate immune processes and intestinal inflammation. Further, chronic inflammation has been shown to increase the risk of development of various cancers including colorectal cancer. Cytokines, as vital they are in inflammation, play a key role in the mediation and regulation of this tumor promoting inflammation. Tumor necrosis factor-α (TNF-α) is a predominantly pro-inflammatory, pleiotropic cytokine and has been reported to play an important role in the pathogenesis and progression of various cancers through the stimulation of vital tumorogenic processes including malignant cellular proliferation; invasion, angiogenesis, induction of endothelial cell proliferation and modulation of the expression of various pro-angiogenic factors (Leek et al., 1998) besides other tumor promoting processes. Several single-nucleotide polymorphisms (SNPs) in human TNF-α gene have been reported to affect the level of gene expression and subsequently the protein function and among these the TNF-α-308G/A SNP is one of the most common TNF-α SNPs in general populations and the one most extensively studied. In this study, we investigated the role of this functional TNF-α-308G/A SNP in the promoter region of TNF-α gene as a potential colorectal cancer risk factor in a case–control study design with 142 case subjects and 184 control subjects.

Tumor necrosis factor-α (TNF-α) gene is located on short arm of chromosome 6 (region p21.33) and is expressed as a 26 kDa membrane bound protein which through proteolytic cleavage by the enzyme metalloproteinase disintegrin called TNF-α converting enzyme (TACE) produces the final 17 kDa soluble active TNF-α molecule. The regulation of TNF-α expression mostly occurs at the transcriptional level (Raabe et al., 1998). The 5′-flanking region of the TNF-α gene encompassing the promoter site is a regulatory hotspot region that contains multiple potential regulatory sites that include those polymorphic sites that coincide with the DNA motifs to which transcription factors like activator protein-1 and 2 (AP-1 and AP-2) bind. It also contains the cAMP-responsive element, DNaseI hypersensitivity site and sequences similar to the kappa B sequences including the NF-κB consensus sequences found in immunoglobulin and cytokine regulatory elements (Spriggs et al., 1992). The TNF-α-308G/A SNP representing guanine (G) to adenine (A) substitution at − 308 bp position is located within this regulatory hotspot region. The less common TNF-α-308A allele has been associated with higher constitutive and inducible TNF-α expression in comparison to the more common TNF-α-308G allele that is associated with relatively lower TNF-α expression (Abraham and Kroeger, 1999, Wilson et al., 1997). The presence of TNF-α-308A allele has been associated with two to three times more transcriptional activity of the gene in comparison to TNF-α-308G allele. In fact, several studies have associated TNF-α- 308 A allele with increased plasma levels of TNF-α. The exact mechanism behind this considerably higher transcriptional activity of TNF-α-308A allele is not known. However, this polymorphism seems to have a direct effect on transcriptional activity as it has been shown that TNF-α-308A allele is part of an extended MHC haplotype HLA-A1-B8- DR3-DQ2 which is associated with high TNF-α production (Wilson et al., 1997). In addition there is a strong possibility that due to this G to A transition, the transcriptional activators bind with a higher affinity at this polymorphic site that in turn leads to stronger transcriptional activation of the TNF-α gene. It has also been proposed that this SNP alters a transcription factor binding site that in turn affects the binding of other proteins to the − 323 to − 285 composite element, resulting in the formation of an altered composite transcriptional element which is associated with higher transcriptional activity (Abraham and Kroeger, 1999).

Several meta-analyses studies have shown a moderate to strong association of TNF-α-308G/A SNP with an increased risk of and in many cases the progression of various cancers including hepatocellular carcinoma (Feng et al., 2014, Hu et al., 2014), prostate cancer (Ma et al., 2014), oral cancer (Yapijakis et al., 2009), lung cancer (Peng et al., 2012, Shih et al., 2006), cervical cancer (Pan et al., 2012, Zhang and Zhang, 2013), gastric cancer (Gorouhi et al., 2008, Hong et al., 2013, Lu et al., 2010, Zhang et al., 2008), esophageal cancer (Wang et al., 2013), breast cancer (Wang et al., 2011, Yang et al., 2011b) and colorectal cancer (Chen et al., 2013, Min et al., 2014). At the protein level, high plasma levels of TNF-α has been found in cancer patients and these high plasma levels have been associated with a poor prognosis in various cancers (Abrahamsson et al., 1993, Nakashima et al., 1998) including CRC (Balkwill, 2002).Various studies have demonstrated the role of TNF-α as a key player in the progression of human CRC (Balkwill, 2002, Sharma et al., 2008). Further, high plasma cytokine levels including that of TNF-α have been associated with the prediction of clinical outcome in advanced stages of CRC (Sharma et al., 2008). All these and several other studies along with the fact that the TNF-α-308G/A SNP can have a direct bearing on the expression pattern of TNF-α protein coupled with the lack of any such previous study in our population provided us an impetus to undertake this case control study.

In this study, we evaluated the differences in the distribution of gender, age, dwelling and smoking habits among the case group and control group subjects and found that these characteristics were not significantly different between these two groups and thus they were not associated with CRC risk. We also evaluated the distribution of the genotypes of TNF-α-308G/A SNP in CRC patients and controls, and found that this TNF-α promoter polymorphism was not significantly associated with the overall modulation of risk of CRC. These results are in accordance with many several previous meta-analyses and other studies that reported no association between the TNF-α-308G/A SNP and colorectal cancer susceptibility (Chen et al., 2013, Landi et al., 2003, Ohtani et al., 2009, Park et al., 1998, Stanilov et al., 2014, Theodoratou et al., 2012, Theodoropoulos et al., 2006, Wang et al., 2011). Further, apart from the wild genotype (GG), we only found the heterozygous genotype (GA) in our population. The variant genotype (AA) was nonexistent in both the case group and control group subjects. The nonexistence of variant genotype (AA) in population based controls that we used in our study in addition to hospital-based controls is important as it represents an actual genotype frequency in our population in comparison to hospital-based controls which may not represent the actual scenario. The lack of TNF-α-308G/A SNP association with CRC risk in the population under study can have many possible reasons. One of the possible reasons may be the absence of the variant genotype (AA) in our population which leads to the *haploinsufficiency effect* whereby the elevated CRC risk associated with homozygous variant (AA) genotype may be significantly decreased when only heterozygous (GA) genotype is present. At the protein level this means that TNF-α expression associated with heterozygous (GA) genotype though will be high compared to wild genotype (GG) but this increased expression resulting from a single TNF-α-308A allele might be insufficient to influence the CRC risk. It should be noted, however, that this *haploinsufficiency effect* though seems plausible here does not stand true for this SNP or any other SNP in every population studied as the final outcome may be influenced by several other intricate factors. Another plausible explanation of this lack of association with CRC risk is related to the location of TNF-α gene within the human leukocyte antigen (HLA) gene complex. TNF-α-308G/A SNP is located in the major histocompatibility complex (MHC) class III, telemetric to the MHC class II and centrometric to MHC class I gene. This is the most polymorphic region of the genome and there is strong linkage disequilibrium (LD) between the alleles across the HLA gene complex including that between TNF-α and other HLA genes. The direct effect of this linkage disequilibrium is that TNF-α gene and the polymorphisms within may also be linked to another gene, region or haplotype. As a result, the association or disassociation of TNF-α haplotypes itself and of other HLA haplotypes with TNF-α phenotypes and further with various pathological conditions might be due to the variation in a linked gene that regulates the expression of this cytokine rather than due to polymorphism within the TNF-α gene itself. Further, due to the wide differences in the distribution of HLA alleles across different populations in different geographical areas, the associations between TNF-α polymorphisms including TNF-α-308G/A SNP and various pathological conditions including cancer shows a considerable variation which makes it important to evaluate and provide a direct functional link of any polymorphism with the disease or condition under study so as to nullify the possibility of observed association or disassociation due to the existence of linkage disequilibrium with the actual causative gene. Therefore, further mechanistic studies are required to fully evaluate and validate the effect of this SNP on CRC risk in our population. The nonexistence of variant genotype (AA) in our population can be explained through the wide variation in the distribution of TNF-α-308A allele across different ethnically defined populations and geographical areas worldwide. The frequency of TNF-α-308A allele is significantly lower in the Asian population compared to most other world populations. The TNF-α-308A is present in 30% of white Caucasians in the United Kingdom, 10–23% of Europeans, 8–10% of South Americans, 5% of South Africans and 2–9% of East Asians (Conway et al., 1997, Cuenca et al., 2001, Keatings et al., 2000, Mastana and Sokol, 1998). This variation is due to the genetic variability at multiple loci which are present widely across different ethnically defined populations throughout the world.

In the present study, we also evaluated the possible effect modulation of CRC risk by age, gender and smoking status. The reason for such an evaluation comes from various studies that have shown that the susceptibility to CRC is significantly affected by age, gender and smoking (Otani et al., 2003). However, we found no significant effect modulation of CRC risk by wild and variant TNF-α-308G/A SNP genotypes in presence of these different possible risk factors. Further studies with large sample size may help to explain this finding.

We also evaluated the association of TNF-α-308G/A SNP with the subsets of various characteristics of the case group subjects under study i.e. age, gender, dwelling, smoking status, tumor location, tumor grade and lymph node status. However, we found no significant association between the subsets of any of these characteristics and the genotypic status of the TNF-α-308G/A SNP. In other words, we found no correlation between these characteristics and the modulation of CRC risk.

The major strengths of this study are the use of histopathologically confirmed CRC samples, involvement of population based controls in addition to hospital-based controls and adjustment of the results for multiple potential confounding (third) variables. The major limitations of this study are modest sample size of the study to detect gene-gene and gene-environment interactions which usually requires much larger sample size. Further studies incorporating a larger sample size and/or another ethnic population in our study are needed to confirm in depth the role of TNF-α-308G/A SNP in relation CRC susceptibility. However, these limitations are unlikely to affect the final outcome of this study.

## 6 Conclusion

We have demonstrated through this study that there is no significant association between the TNF-α-308G/A promoter single-nucleotide polymorphism and the risk of developing CRC in our ethnic Kashmiri population. Further, we have also shown that there is no significant association between this SNP and various clinico-pathological parameters, demographic variables and environmental factors. However, it is important to emphasize that this study does not nullify the role of TNF-α itself in the pathogenesis of CRC but only indicates that this particular and important TNF-α SNP may not be associated with CRC risk in the population studied. Further, our findings need to be replicated with bigger sample size and should involve other ethnically defined populations with high CRC risk.

## Conflict of interest

None exist between any parties.

## Author disclosure statement

MZB acknowledges University Grants Commission (UGC), GOI for UGC-BSR fellowship.

## Figures and Tables

**Table 1:** Various clinico-pathological parameters, demographic variables and environmental factors in colorectal cancer case subjects and relevant parameters in control subjects from Kashmir.

Characteristics	Colorectal cancer cases (N = 142)^a^	Controls (N = 184)^a^	Pearson χ^2^; p value
*Age (years)*			
Mean age (SD) (SEM)^b^	52.68 (15.34) (1.29)	52.22 (14.57) (1.07)	
Age range (median)	21–82 (55)	21–80 (51.5)	
≤ 50	66 (46.48%)	91 (49.46%)	0.29; 0.59
> 50	76 (53.52%)	93 (50.54%)

*Gender*			
Male	85 (59.86%)	102 (55.43%)	0.64; 0.42
Female	57 (40.14%)	82 (44.57%)

*Place of residence*			
Rural	87 (61.27%)	101 (54.89%)	1.33; 0.25
Urban	55 (38.73%)	83 (45.11%)

*Smoking status*			
Ever	80 (56.34%)	94 (51.09%)	0.89; 0.35
Never	62 (43.66%)	90 (48.91%)

*Tumor location*			
Colon	58 (40.85%)		
Rectum	84 (59.15%)		

*Tumor grade*			
W.D.	95 (66.90%)		
M.D. and P.D.	47 (33.10%)		

*Lymph node status*			
Involved	78 (54.93%)		
Not involved	64 (45.07%)		

Pearson chi-square test (χ^2^) was used to calculate the p values for categorical variables.

a N denotes number of subjects or individuals.

b SD and SEM stand for standard deviation and standard error of mean respectively.

**Table 2:** TNF-α-308G/A single nucleotide polymorphism genotype frequency distributions among CRC cases and matched controls and risk of CRC.

	CRC cases (N = 142)*	Controls (N = 184)*	Odds ratio (95% CI); Fisher p value^#^	Adjusted odds ratio^1^ (95% CI); Fisher p value^#^	χ^2^; Pearson p value (overall)^#2^
*Genotype*					
GG	124 (87.32%)	150 (81.52%)	1.0 (Reference)	1.0 (Reference)	2.01; 0.156
GA	18 (12.68%)	34 (18.48%)	1.51 (0.80–2.87); 0.207	1.56 (0.82–2.95); 0.178
AA	0 (0%)	0 (0%)		
GA + AA	18 (12.68%)	34 (18.48%)	1.51 (0.80–2.87); 0.207	1.56 (0.82–2.95); 0.178	2.01; 0.156

*Allele*					
G	266 (93.66%)	334 (90.76%)	1.0 (Reference)		
A	18 (6.34%)	34 (9.24%)	1.50 (0.83–2.72); 0.191		1.84; 0.175

*N denotes number of subjects or individuals. ^#^The p values in bold indicate significant results. CI, confidence interval; CRC, colorectal cancer; OR, odds ratio. ORs (95% CIs) were obtained from conditional logistic regression models. ^1^Adjusted ORs (95% CIs) was obtained in conditional logistic regression models when adjusted for age, gender, place of residence and smoking status. ^2^P-values calculated using χ^2^-tests.

**Table 3:** Effect modulation of TNF-α-308G/A SNP genotypes in presence of various risk factors of CRC in Kashmir, India.

Genotype^ and characteristic	CRC cases N (%)	Controls N (%)	Odds ratio (95% CI); Fisher p value^#^	Adjusted odds ratio^1^ (95% CI); Fisher p value^#^	χ^2^; Pearson p value (overall)^#2^
*Age*					
Wild and ≤ 50	56 (39.44)	71 (38.59)	1.0 (Reference)	1.0 (Reference)	
Variant and ≤ 50	10 (7.04)	20 (10.87)	1.46 (0.64–3.36); 0.368	1.51 (0.66–3.46); 0.329	2.19; 0.534
Wild and > 50	68 (47.89)	79 (42.93)	1.23 (0.19–8.12); 0.833	1.26 (0.19–8.58); 0.812
Variant and > 50	8 (5.63)	14 (7.61)	1.95 (0.23–16.62); 0.541	2.01 (0.23–17.67); 0.528

*Gender*					
Wild and male	72 (84.71)	84 (82.35)	1.0 (Reference)	1.0 (Reference)	
Variant and male	13 (15.29)	18 (17.65)	1.22 (0.54–2.79); 0.629	1.46 (0.62–3.43); 0.387	0.19; 0.667
Wild and female	52 (91.23)	66 (80.49)	1.0 (Reference)	1.0 (Reference)	
Variant and female	5 (8.77)	16 (19.51)	2.075 (0.72–5.95); 0.174	2.13 (0.73–6.21); 0.168	3.02; 0.082

*Smoking status*					
Wild and non-smoker	57 (40.14)	72 (39.13)	1.0 (Reference)	1.0 (Reference)	
Variant and non-smoker	5 (3.52)	18 (9.78)	2.37 (0.84–6.70); 0.104	2.49 (0.88–7.02); 0.086	4.91; 0.179
Wild and smoker	67 (47.18)	78 (42.39)	0.93 (0.27–3.29); 0.915	1.02 (0.22–4.64); 0.979
Variant and smoker	13 (9.15)	16 (8.70)	0.10 (0.23–4.28); 0.998	1.10 (0.21–5.69); 0.911

^Wild refers to GG genotype and variant refers to GA + AA genotype. *N denotes number of subjects or individuals. ^#^The p values in bold indicate significant results. CI, confidence interval; CRC, colorectal cancer; OR, odds ratio. ORs (95% CIs) were obtained from conditional logistic regression models. ^1^Adjusted ORs (95% CIs) was obtained from conditional logistic regression models when adjusted for age, gender, place of residence and smoking status. The variable under consideration was excluded at the time of analysis. ^2^P-values calculated using χ^2^-tests.

**Table 4:** Association of TNF-α-308G/A polymorphism with various clinico-pathological parameters, demographic variables and environmental factors in CRC cases.^a^

Characteristics	N = 142	XX	XY	YY	χ^2^; p value
**124(87.32%)**	**18(12.68%)**	**0(0%)**
*Age (years)*					
≤ 50	66 (46.48%)	56 (45.16%)	10 (55.56%)	0 (%)	0.68; 0.41
> 50	76 (53.52%)	68 (54.84%)	8 (44.44%)	0 (%)

*Gender*					
Male	85 (59.86%)	72 (58.06%)	13 (72.22%)	0 (%)	1.31; 0.25
Female	57 (40.14%)	52 (41.94%)	5 (27.78%)	0 (%)

*Dwelling*					
Rural	87 (61.27%)	74 (59.68%)	13 (72.22%)	0 (%)	1.04; 0.31
Urban	55 (38.73%)	50 (40.32%)	5 (27.78%)	0 (%)

*Smoking status*					
Ever	80 (56.34%)	67 (54.03%)	13 (72.22%)	0 (%)	2.11; 0.14
Never	62 (43.66%)	57 (45.97%)	5 (27.78%)	0 (%)

*Tumor location*					
Colon	58 (40.85%)	53 (42.74%)	5 (27.78%)	0 (%)	1.46; 0.23
Rectum	84 (59.15%)	71 (57.26%)	13 (72.22%)	0 (%)

*Tumor grade*					
W.D.	95 (66.90%)	83 (66.94%)	12 (66.67%)	0 (%)	0.99; 1
M.D. and P.D.	47 (33.10%)	41 (33.06%)	6 (33.33%)	0 (%)

*Lymph node status*					
Involved	78 (54.93%)	66 (53.23%)	12 (66.67%)	0 (%)	1.15; 0.28
Not involved	64 (45.07%)	58 (46.77%)	6 (33.33%)	0 (%)

a The values in bold, if any indicate significant results. The abbreviations OR, WD, MD and PD denote odds ratio, well differentiated, moderately differentiated and poorly differentiated respectively.
